# Progress in Surgery

**Published:** 1900-05-12

**Authors:** 


### PROGRESS IN SURGERY.

### DISEASES OF THE BLADDER AND PROSTATE GLAND.

(*Continued from page* 86).

**Prostatectomy and Bottini's Operation.**—These procedures were discussed at the meeting of the New York Academy of Medicine on December 11th, 1899.[5] A. D. Johnson presented a patient, aged 60, upon whom perineal prostatectomy had been performed. The man had suffered for 50 years from difficult, painful, and frequent micturition. There were regularly five ounces of residual urine. The bladder was opened above the pubes. The prostate was reached through a perineal incision, its capsule incised on either side, and each half enucleated with the finger. The median portion came away easily. Mesially the mucous membrane was torn where attached to the gland. The bleeding was free. A soft rubber catheter was inserted through the rent in the urethra into the bladder, and the cavity left packed with gauze. After the seventh day a catheter was passed daily. At the end of two months the patient had gained 25 pounds in weight. The quantity of residual urine had much diminished. Faradisation of the bladder had contributed to the latter result. Forbes Hawkes presented a man of 70, upon whom he had successfully performed supra-pubic prostatectomy. The man had had several attacks of retention previous to the operation, and there had been difficulty in catheterisation. At the present time he slept ten hours without urinating. Willy Meyer demonstrated Frendenberg's modification of Bottini's prostatic cauterisator. He expressed the firm conviction that Bottini's operation had come to stay. The operation might enable the surgeons to get their patients at an early stage of the disease. Bolton Bangs knew of cases in which Bottini's operation had absolutely failed, and he was endeavouring to find principles which would guide surgeons in the proper selection of cases for this operation. He thought more success was attained in the soft adenomatous type of enlargement. Samuel Alexander pointed out that one important cause of vesical retention in these cases was the displacement of the insertion into the prostate of the vesical muscle. As a result the regular work of contraction of the detruser was interfered with, resulting in partial retention. It would appear that theoretically an operation deserving the name "radical operation for the relief of prostatic enlargement" must contemplate the removal of the mechanical obstruction, the relief of the venous stasis, the restoration to nearly its normal position of the attachment of the vesical muscle to the prostate, and the relief of congestion and inflammation of the bladder by drainage. Prostatectomy was the only operation which did this. The Bottini cautery solely removed the mechanical obstruction caused by the growth, and it did that only partially. He had seen several cases which had been operated upon by this method by well-known surgeons, and in all the ultimate results had been unsatisfactory. Too much had been claimed for the operation. Speaking of the technique of perineal prostatectomy, Dr. Alexander said in most cases a supra-pubic incision into the bladder was necessary in order to bring the prostate down into the perineum. He had employed the longitudinal incision in the perineum more than the transverse. The former healed more rapidly, and was just as effective. He then usually divided the floor of the membranous urethra up to the apex of the prostate. Then he broke through the mucous membrane of the prostatic urethra as near the apex as possible, coming at once upon the enlarged lateral lobe, and being able to separate this without difficulty from the prostatic capsule. The mucous membrane undoubtedly became lacerated below the veru montanum, but the mucous membrane above that structure was the portion it was necessary to preserve intact. Bottini,[6] lecturing on prostatic ischuria, described two objects with which the cautery was used, viz., for simple cauterisation, and for incision by the cautery. The indications for the two operations were different, and that of incision had the wider application. The indications for simple cauterisation were then given. They were as follows: (1) Enlargement of the first degree in the supra-montanal region or median lobe; (2) enlargement of one lateral lobe with initial median enlargement; (3) enlargement of the median lobe with engorgement of the prostatic mucous membrane. It was also useful in spasm of the neck of the bladder, and in certain forms of chronic cervical cystitis. A 1 per cent. solution of cocaine was satisfactory as an anæsthetic. The eschar begins to separate about the eighth day. The good effects are shown usually about the twentieth to the twenty-eighth day, when the separation of the eschar is complete.

[5] Med. Rec., Dec., 1899. [6] Brit. Med. Jour., Epit. Jan., 1900.

